# Cholinergic Deficit Induced by Central Administration of 192IgG-Saporin Is Associated With Activation of Microglia and Cell Loss in the Dorsal Hippocampus of Rats

**DOI:** 10.3389/fnins.2019.00146

**Published:** 2019-03-12

**Authors:** Yulia V. Dobryakova, Maria N. Volobueva, Anna O. Manolova, Tatiana M. Medvedeva, Alexey A. Kvichansky, Natalia V. Gulyaeva, Vlamidir A. Markevich, Mikhail Yu. Stepanichev, Alexey P. Bolshakov

**Affiliations:** ^1^Institute of Higher Nervous Activity and Neurophysiology, Russian Academy of Sciences, Moscow, Russia; ^2^Research Laboratory of Electrophysiology, Pirogov Russian National Research Medical University, Moscow, Russia

**Keywords:** 192IgG-saporin, dorsal hippocampus, microglia, vascular endothelium, CA3 area

## Abstract

Alzheimer’s disease (AD) is associated with degeneration of cholinergic neurons in the basal forebrain. Administration of the immunotoxin 192IgG-saporin to rats, an animal model of AD, leads to degeneration of cholinergic neurons in the medial septal area. In the present study, cholinergic cell death was induced by intracerebroventricular administration of 192IgG-saporin. One and a half months after injection, we studied the histopathology of the hippocampus and the responses of microglia and astrocytes using immunohistochemistry and neuroglial gene expression. We found that treatment with 192IgG-saporin resulted in neuronal loss in the CA3 field of the hippocampus. Microglial proliferation was observed in the dentate gyrus of the dorsal hippocampus and white matter. Massive proliferation and activation of microglia in the white matter was associated with strong activation of astrocytes. However, the expression of microglial marker genes significantly increased only in the dorsal hippocampus, not the ventral hippocampus. These effects were not related to non-specific action of 192IgG-saporin because of the absence of the Nerve growth factor receptor in the hippocampus. Additionally, 192IgG-saporin treatment also induced a decrease in the expression of genes that are associated with transport functions of brain vascular cells (Slc22a8, Ptprb, Sdpr), again in the dorsal hippocampus but not in the ventral hippocampus. Taken together, our data suggest that cholinergic degeneration in the medial septal area induced by intracerebroventricular administration of ^192^IgG-saporin results in an increase in the number of microglial cells and neuron degeneration in the dorsal hippocampus.

## Introduction

Impairment of cholinergic neurotransmission is a characteristic feature of different neurodegenerative diseases including Alzheimer’s disease (AD) (Hampel et al., 2018), Parkinson’s disease with dementia, and dementia with Lewy bodies (DLB) (Liu et al., 2018). AD is predominantly associated with the death of cholinergic neurons in the nucleus basalis of Meynert (NBM) (Mufson et al., 1989; Fujishiro et al., 2006) and, in some cases, with the degeneration of cholinergic neurons in the medial septum and diagonal band of Broca (DBB) area (Lehéricy et al., 1993). Parkinson’s disease with dementia is also associated with a decrease in the number of cholinergic neurons in the NBM but not in the medial septum and DBB area (Hall et al., 2014). The DLB is associated with a loss of cholinergic neurons in both the NBM and the medial septum and DBB area (Fujishiro et al., 2006). Taken together, current data suggest that cholinergic deficit is one of the key points in the development of different neurodegenerative diseases. One widely used approach for the analysis of the consequences of cholinergic degeneration in the brain is induction of selective death of cholinergic neurons by the immunotoxin 192IgG-saporin (Ig-saporin), which is injected either intracerebroventricularly or intraseptally (Wiley et al., 1991; Heckers et al., 1994; Schliebs et al., 1996; Potter et al., 1999). Ig-saporin is a conjugate of an antibody against the nerve growth factor receptor (NGFR, p75 in old nomenclature) and the ribosomal toxin saporin, which after penetration into the cell inactivates ribosomes and arrests protein synthesis, leading to cell death (Wiley et al., 1991; Book et al., 1995). Ig-saporin selectively eliminates cholinergic neurons in the septum and DBB area after either intraseptal or intracerebroventricular injection and does not affect other neuronal populations in these regions (Heckers et al., 1994; Johnson et al., 2002).

The death of cholinergic neurons in the medial septum and DBB is associated with the development of weak behavioral deficits in various behavioral tasks (Janisiewicz et al., 2004; Paban et al., 2010; Jeong et al., 2014; Knox and Keller, 2016; Dobryakova et al., 2017). Analysis of gene expression after Ig-saporin administration revealed changes in the expression of a large number of genes in the hippocampus (Paban et al., 2010); a more detailed analysis showed that intracerebroventricular administration of Ig-saporin predominantly affects gene expression in the dorsal but not ventral hippocampus (Dobryakova et al., 2017). Interestingly, the majority of genes, whose expression increased ∼1.5 months after injection of Ig-saporin in the dorsal hippocampus, were genes that are predominantly expressed by microglial cells. This suggests that Ig-saporin, when injected intracerebroventricularly, may lead to the activation and/or proliferation of microglia in the dorsal part of the hippocampus. According to previous data, intracerebroventricular (i.c.v). administration of Ig-saporin results in microglia activation in both the septum and hippocampus 7 days after injection of the immunotoxin (Rossner et al., 1995a,b; Seeger et al., 1997). However, the long-term consequences and pattern of changes that occur in the hippocampus and adjacent structures after Ig-saporin are not known. Our RNA-seq data (Dobryakova et al., 2017) suggest that upregulation of microglia functioning may still exist in the dorsal hippocampus even 1.5 months after Ig-saporin administration, suggesting that Ig-saporin induced some long-term changes in the state of microglia in the hippocampus.

It has been shown in a number of studies that activation of microglia may lead to neural cell death (Fan et al., 2015). The aforementioned findings suggest that Ig-saporin-induced microglia activation in the dorsal hippocampus may cause delayed neuronal death in this structure; however, so far neuronal survival in the hippocampus has not been evaluated at a postponed time period after lesion of the cholinergic neurons of the medial septum. Therefore, the aim of the present study was to analyze the postponed effect of cholinergic deficit induced by Ig-saporin on the functional state of microglia and neurodegenerative processes in the hippocampus.

## Materials and Methods

The experiments were performed with adult male Sprague-Dawley rats (250–350 g) taken from “Pushchino” nursery (Shemyakin and Ovchinnikov Institute of Bioorganic Chemistry, Russian Academy of Sciences). A total of 25 rats were involved in the study (control *n* = 12; Ig-saporin *n* = 13). Animals were housed under standard vivarium conditions at 21 ± 1°C with a 12 h light/dark cycle; food and water were provided *ad libitum*. All experiments were performed in accordance with the ethical principles stated in the EU Directive 2010/63/EU for animal experiments and were approved by the Ethical Committee of the Institute of Higher Nervous Activity and Neurophysiology of the Russian Academy of Sciences.

### Injection of Ig-Saporin

Rats were anesthetized with chloral hydrate (400 mg/kg, i.p.). 192IgG-saporin (Millipore Corporation, Temecula, United States; 1 μg/μl) was injected bilaterally into both ventricles (i.c.v.) at a dose of 4 μg/site, which induced a strong loss of cholinergic neurons in the septum (Supplementary Figure S1). The bilateral injections were performed stereotaxically into the lateral ventricles (0.8 mm posterior, 1.5 mm lateral to bregma) (Paxinos and Watson, 1998) using a 10 μl Hamilton syringe (Hamilton, Giarmata, Romania). Control rats received an equal volume of sterile PBS. Rats were allowed to recover for 1.5 months before morphological analysis. In control (*n* = 7) and Ig-saporin-treated (*n* = 7) animals, one hemisphere of the brain was taken for morphological analysis and the other hemisphere was taken for analysis of gene expression; in the other animals (control *n* = 6; Ig-saporin *n* = 5), the entire brain was taken for morphological analysis.

### Immunohistochemistry

Rats were anesthetized with chloral hydrate (400 mg/kg, i.p.) 1.5 months after the injection and then submitted to transcardial perfusion with ice-cold 0.9% NaCl. For morphological analysis, either the entire brain or one hemisphere was removed and postfixed in 4% paraformaldehyde (Sigma-Aldrich, United States) solution on PBS (PBS, Biolot, Russia) for at least 2 days.

The septum and dorsal hippocampus were sectioned into 50-μm-thick coronal brain sections using a vibratome (Leica VT1200 S). Six sections, each of which included the medial septal nucleus from each rat, were selected and stained for choline acetyltransferase (ChAT) as previously described (Dobryakova et al., 2017). In brief, sections were incubated in 0.3% Triton X-100 (SERVA, Germany) in 0.01 M PBS (PBS-T) three times for 5 min at room temperature, then for 1 h in blocking solution consisting of 5% normal goat serum (Sigma-Aldrich, United States) in PBS-T and then in blocking solution with primary antibodies (rabbit anti-ChAT 1:500, Santa Cruz Biotechnology, United States) at 4°C overnight. The next day, the sections were washed in PBS-T and incubated with secondary antibodies (1:800, goat anti-rabbit IgG-biotin, Sigma-Aldrich, United States) in blocking solution at room temperature for 1 h. After additional washing in PBS, the sections were incubated with an avidin–biotin–HRP complex (ABC Elite kit, Vector Labs, United States) for 1 h, and 3,3′-diaminobenzidine (SIGMA-Fast Kit, Sigma-Aldrich, United States) was used as a chromogen for staining. All images were acquired with a Keyence 6000EZ microscope (Keyence, Japan). Imaging parameters were set to avoid signal saturations. Typically, Ig-saporin caused a strong loss of ChAT-positive neurons in the septal area (Supplementary Figure S1). To evaluate the death of ChAT-positive cells in the medial septum and the DBB, we counted the number of ChAT-positive cells in the area of the medial septum and DBB as described previously (Dobryakova et al., 2017). Animals, in which Ig-saporin-induced death of ChAT-positive neurons was over 50% compared to the mean value of control rats, were taken for further analysis.

Six sections from the hippocampus area from each rat were selected and stained for IBA-1 and glial fibrillary acidic protein (GFAP). Double immunofluorescence labeling of Iba-1 and GFAP was performed using co-incubation with the respective primary antibodies. The floating sections were first incubated in PBS-T three times for 5 min, then in blocking solution (5% normal donkey serum (Sigma-Aldrich, United States) in PBS-T) for 1 h and then in blocking solution with a mixture of primary antibodies (rabbit anti-IBA-1 1:400, Wako, Japan; mouse monoclonal anti-GFAP-Cy3 conjugate 1:400, Sigma-Aldrich, United States) at 4°C overnight. The next day the sections were washed and incubated with the Alexa Fluor-488 conjugated donkey anti-rabbit IgG (Life Technologies, United States). Sections were coverslipped with Prolong Gold with DAPI mounting medium (Thermo Fisher Scientific, United States) and stored at 4°C.

### Microglia Cell Counting

All images were acquired with a Keyence 6000EZ microscope. Imaging parameters were set to avoid signal saturations. Images of the dentate gyrus and CA3 and CA1 areas of the dorsal hippocampus, and the white matter which is located between the dorsal hippocampus and neocortex, which predominantly includes the corpus callosum were taken. The images of hippocampal areas included the hilus in the dentate gyrus and the stratum radiatum in the CA1 and CA3 areas.

Image processing and quantitative analysis of microglial morphology changes were performed using ImageJ/Fiji software (Version 1.51, NIH, United States). The maximum intensity projection function was used to get a 2D image from the z-stack images obtained from the microscope (40×/0.95 lens). Since cell soma is the brightest object on the microphotograph and the background was evenly lit, we used global thresholding. The steps of processing were as follows: converting to 8-bit; enhancing contrast; applying default threshold. After that, all objects greater than 300 pxls were counted using the “Analyze particles...” function.

### Nissl Staining and Counting of Neurons

Three sections from the dorsal hippocampus were selected using an unbiased random sampling scheme with a distance of 300 um between them. The sections were mounted onto gelatin-coated slides and Nissl-stained using 0.1% cresyl violet (Merck, United States). The sections were dehydrated and coverslipped with Histofluid (Marienfeld, Germany).

Cells with neuronal morphology were counted in one optical section using an Olympus CX-41 microscope (Olympus, Japan) at ×400 magnification. Only cells with clearly visible nuclei and nucleoli were counted using a 250 um × 250 um eyepiece grid. The grid was randomly placed over the pyramidal layer of CA1, CA3 or hilus of the hippocampus. The number of cells within the grid was counted in the left and right hemispheres of the hippocampus and summed for each of the stained sections. The mean value from the three sections was assumed as the number of cells in the region of interest of the animal. Cell densities in the CA1 or CA3 field of the hippocampus as well as ectopic cell density in the stratum lucidum of the CA3 are presented as number of cells per mm^2^.

### RNA Isolation

After brain sampling, fragments of gray matter from the parietal lobe of the neocortex, septal third and temporal quarter of the hippocampus were collected. The septal and temporal parts of the hippocampus were considered as the dorsal and ventral parts, respectively (Moser and Moser, 1998). Total RNA was extracted from the brain samples and dissolved in ExtractRNA (Evrogen, Moscow, Russia) following the manufacturer’s protocol. Total RNA concentration was measured using a NanoDrop 2000 spectrophotometer (Thermo Fisher Scientific, United States). To remove traces of genomic DNA, 1 μg total RNA was treated with DNase I (Thermo Fisher Scientific, United States).

### cDNA Synthesis

First strand cDNA were synthesized using a Reverse transcriptase MMLV kit (Evrogen, Moscow, Russia) with random decaprimers according to the manufacturer’s instruction.

### Quantitative Real-Time PCR

RT-qPCR was performed using a 7500 Real-Time PCR System (Applied Biosystems, United States). The temperature profile was (1) 95°C for 10 min, (2) 40 cycles of 95°C for 15 s, 63°C for 30 s, and 72°C for 30 s, and (3) melt curve analysis with measures between 60 and 95°C. HS-SYBR+ROX (Evrogen, Moscow, Russia) was used to prepare the reaction mixes.

The Il1β expression in the hippocampus is close to the limit of qPCR sensitivity; hence, to quantitatively compare samples, the remaining cDNA was concentrated using isopropanol. The precipitate was washed with 80% ethanol, diluted in 10 μl deionized water, and then analyzed using qPCR.

Gene expression was calculated as relative quantity RQ = 2^ΔΔCt^; the expression was normalized to the expression of the gene Trappc10, which, according to our previous transcriptomic data, is one of the genes with the most stable expression under the studied conditions (Dobryakova et al., 2017). Expression of all genes was normalized to an external reference sample.

### Primer Design and Validation

Primers for qRT-PCR analysis were designed using Primer Select (Table 1). The primers were synthesized by Evrogen (Moscow, Russia). Target specificity was examined by melt curve analysis and 2% agarose electrophoresis. Efficiency of amplification was evaluated using serial dilutions (1:1, 1:4, 1:16, 1:64, 1:256).

**Table 1:** List of primers and their characteristics.

Gene	Pubmed reference	Primer sequence	Product length, bp	Efficacy
*IL1β*	NM_031512.2	F-TCT GTG ACT CGT GGG ATG AT R- CAC TTG TTG GCT TAT GTT CTG TC	161	100
*Trappc10*	NM_001173528. 1	F-TGG TCA TTC CCA GTC AAG ACG R-TGA GCA GCT GAA GGA CAC TAT TCT	206	84
*Ncf1*	NM_053734.2	F-CTG CAG CAA AGG ACA GGA CTG R-GGG TCA TGG CCA ACA GGT T	202	91
*Cx3Cr1*	NM_133534.1	F-GGA CCT CAC CAT GCC TAC CT R-CAC CAA CAG ATT CCC CAC CAG	169	100
*Ptprb*	XM_008765424. 2	F-GAC TCC GAC TTC GAT GGC TAC A R-CGG TCT ATC ATG GTG ATG GTG C	206	86
*Slc22a8*	NM_031332.1	F-GGT GGC TAC CTT CAA CGG C R-TGT GAT GAA CTT GGC TGG GAT G	286	92
*Slc2a5*	NM_031741.1	F-GGC CTC ATC TTC CCG TTC AT R-GCC TGG CTC TGC TAC TGC TC	244	82
*Sdpr*	NM_001007712. 1	F-ACG AAT TGC CCG GTG ATG AGG R-GGT GGT TGG GAG TGA GCG ATT TCT	222	100


## Results

### The Effect of Ig-Saporin on the Morphology of Glia in the Hippocampus and White Matter

**FIGURE 1:**
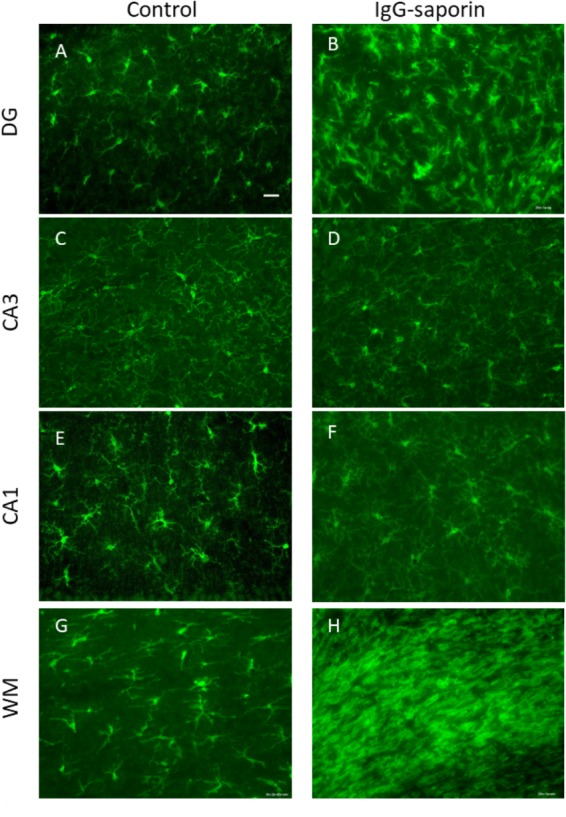
Effect of Ig-saporin on morphology of microglia in the dentate gyrus (DG), hippocampal CA1 and CA3 areas, and the white matter. Sections of the dorsal hippocampus of rats intracerebroventricularly treated with PBS **(A,C,E,G)** or Ig-saporin **(B,D,F,H)** were stained using anti-IBA1 antibodies. Microglial cells have thicker and shorter processes and their number increased in the DG, CA3, and white matter as compared to the control 1.5 months after Ig-saporin injection. Scale bar, 40 μm.

**FIGURE 2:**
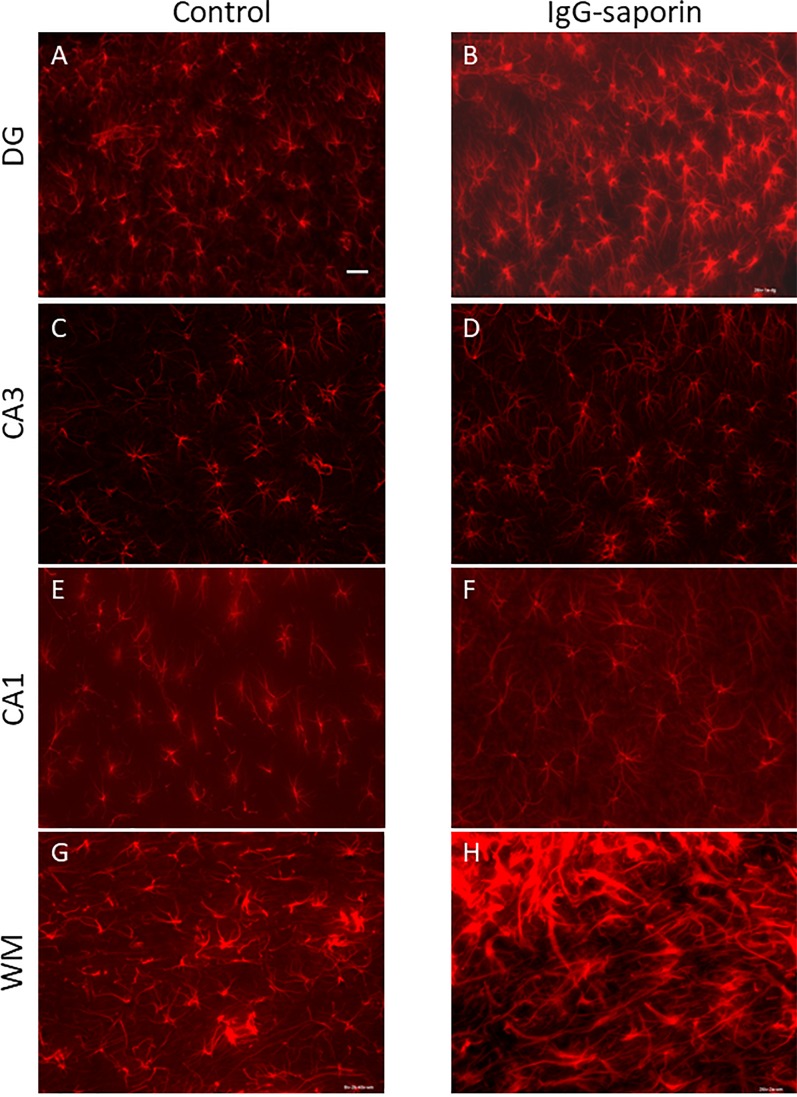
Effect of Ig-saporin on the morphology of astrocytes in the dentate gyrus (DG), hippocampal CA1 and CA3 areas, and white matter. Sections of the dorsal hippocampus of rats intracerebroventricularly treated with PBS **(A,C,E,G)** or Ig-saporin **(B,D,F,H)** were stained using anti-GFAP antibodies. There is no effect on astrocyte morphology 1.5 months after Ig-saporin injection in DG **(A,B)**, CA3 **(C,D)**, and CA1 **(E,F)** areas. In contrast, in animals, where we observed massive microgliosis (Figure 1H), we also observed activated astrocytes which had increased soma size and strong thick processes **(G,H)**. Scale bar, 40 μm.

Under control conditions, microglial cells in all areas studied [dentate gyrus (DG), CA1 and CA3 areas] had small bodies and thin ramified processes (Figure 1), i.e., microglial morphology that is typical of resting cells. In addition to the hippocampus, we also analyzed the state of microglia in the white matter localized between the neocortex and hippocampus (Figure 1G,H). In all the areas studied, astrocytes also had the appearance of resting non-activated cells with small somas and thin ramified processes (Figure 2).

**FIGURE 3:**
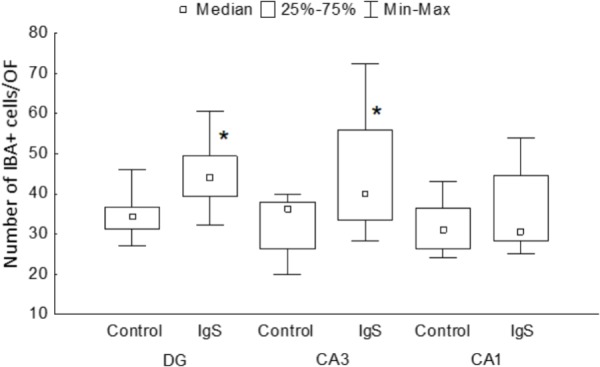
Statistical data on the number of IBA-positive cells in the hippocampal areas. DG, dentate gyrus; IgS, 192IgG-saporin; ^∗^significant differences compared to control (for details, see text).

One and a half months after injection of the immunotoxin, we observed a significant increase in the number of IBA-positive cells in the DG (significant difference between control (*n* = 13) and Ig-saporin (*n* = 12), Kruskal-Wallis ANOVA, *p* = 0.0033) (Figure 1, 3). An increase in the number of microglial cells in the DG was associated with a thickening of microglial processes and a shortening of outgrowths reflecting activation of microglia.

In the CA1 area, we did not observe any effect of Ig-saporin on the number of microglial cells (Figure 3), however, in the CA3 area the effect of the immunotoxin varied among animals. In all animals, Ig-saporin induced a significant increase in the number of microglial cells (Kruskal-Wallis ANOVA, *p* = 0.036; Figure 3); however, this increase in the majority of animals (*n* = 10) was associated with minor changes in the morphology of microglial cells (Figure 1C,D) whereas in the rest of the animals (*n* = 2) we observed swelling of cells and substantial shortening of their processes (Supplementary Figure S2). These changes in the microglial morphology corresponded to the state of astrocytes in the CA3 area: in animals where Ig-saporin did not affect microglial morphology, we also did not observe any changes in the astrocyte morphology, whereas strong microgliosis in CA3 was associated with swelling of astrocytes and thickening of their processes (Figure 2C,D and Supplementary Figure S2).

Analysis of the state of white matter unexpectedly showed that in 7 out of 13 animals, Ig-saporin also induced massive gliosis in the white matter (Figure 1, 2) whereas in the remaining rats it had no visible effect. The gliosis produced by Ig-saporin was so dense that it was hard to identify single cells and count them (Figure 1). As for astrocytes in the white matter, we also observed strong changes in their morphology; microgliosis was associated with an increase in the body size of astrocytes and thickening of their processes (Figure 2). The latter suggests that Ig-saporin also induced activation of astrocytes in these animals.

### The Effect of Ig-Saporin on the Expression of Microglial and Vascular Markers in the Hippocampus

**FIGURE 4:**
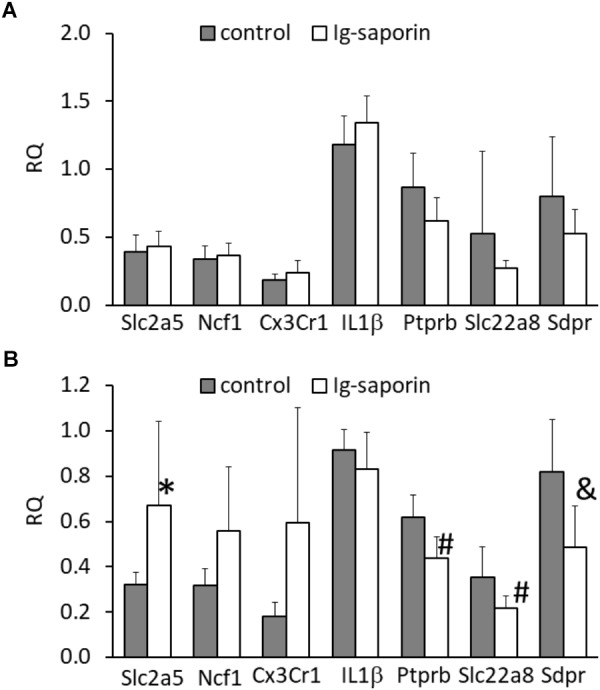
Effect of intracerebroventricular administration of Ig-saporin on the expression of microglial markers (Slc2a5, Ncf1, Cx3Cr1), Il1β, and vascular markers (Ptprb, Slc22a8, Sdpr) in the ventral **(A)** and dorsal **(B)** hippocampus. The data are presented as mean ± SD. ^∗^Significant difference compared to control (*p* = 0.005, Mann-Whitney *U-*test). ^#^Significant differences compared to control (*p* < 0.008, Mann-Whitney *U*-test); ^&^significant differences compared to control (*p* = 0.011, Mann-Whitney *U*-test).

Next, we analyzed whether the changes in microglial state that we observed at the morphological level are associated with changes at the molecular level. Analysis of gene expression in the dorsal and ventral parts of the hippocampus showed that the ventral hippocampus was not affected by treatment with Ig-saporin (Figure 4A) whereas in the dorsal hippocampus the immunotoxin induced some changes in the expression of genes. First, we found that the expression of the microglial marker Slc2a5 was significantly increased and the expression of the microglial markers Ncf1 and Cx3Cr1 also tended to be elevated (*p* = 0.06 and *p* = 0.08, respectively, Mann-Whitney *U*-test). The expression of Il1β was similar to that in the control.

Our next question was whether Ig-saporin induced any changes in the dorsal hippocampus except microglial activation and neuronal death. Inflammation typically involves changes in the functioning of blood vessels (Akanuma et al., 2011), so we analyzed whether Ig-saporin affected expression of vessel-related genes. To examine the state of brain vascular cells, we selected three genes – Ptprb, Slc22a8, and Sdpr – that are selectively expressed in vascular cells, such as pericytes and endothelium (Zeisel et al., 2015, 2018; Vanlandewijck et al., 2018), and are associated with transport functions of these cells or regulation of this function. We found that, in the dorsal hippocampus, expression of all these genes decreased (Figure 4B).

### The Effect of Ig-Saporin on Neuronal Survival in the Hippocampus

**FIGURE 5:**
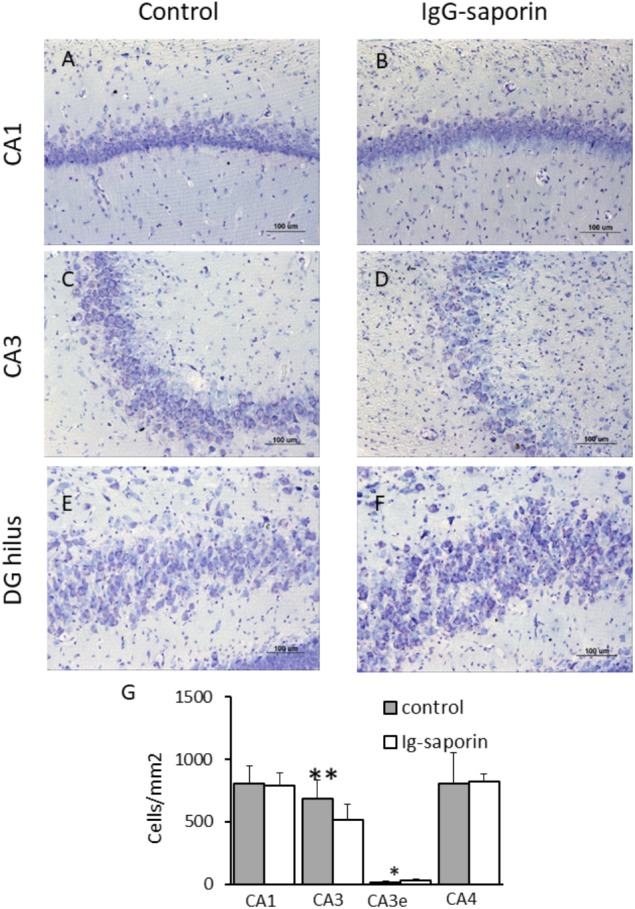
Effect of Ig-saporin on neuronal survival in different hippocampal areas. Brain sections of rats intracerebroventricularly treated with PBS **(A,C,E)** or Ig-saporin **(B,D,F)** were Nissl-stained and the number of neurons was counted as described in the methods. Ig-saporin had no effect on the number of neurons in CA1 **(A,B)** and CA4 **(E,F,G)** areas but induced a significant decrease in the number of CA3 neurons in the pyramidal layer. This decrease was associated with an increase in the number of CA3 pyramidal neurons in the extrapyramidal space (CA3e, panel **E**). ^∗^Significant difference compared to control (*p* = 0.013); ^∗∗^significant difference compared to control (*p* = 0.006).

Nissl staining revealed that most neurons in the main cell layers of the hippocampus of control rats had a normal appearance with clearly visible nuclei, nucleoli, and perinuclear cytoplasm. We found that in the pyramidal layer of the CA1 hippocampal field of the rats subjected to Ig-saporin, most neurons were similar to those observed in the control group and their density did not significantly differ from that found in the control rats. However, in the pyramidal layer of the CA3, cell loss was clearly visible. Multiple cells with pathological alterations including karyolysis, cell swelling and lysis were observed within this area. Calculation of cell density supported this observation [Figure 5; *t*(22) = 3.04, *p* = 0.006; Student’s *t*-test]. Paradoxically, neuronal death in the CA3 area after Ig-saporin was associated with a significant increase in the number of neurons located ectopically in the CA3 stratum lucidum, most of which were dark or shrunken [Figure 5C,D; *t*(22) = –2.7, *p* = 0.013]. Interestingly, we did not find significant differences in the number of neurons located in the hippocampal hilus (CA4 field). Thus, our data indicate that microglia activation, frequently considered as a factor promoting damage to neurons, was associated with significant cell loss and an increase in the number of damaged neurons in the hippocampus.

**FIGURE 6:**
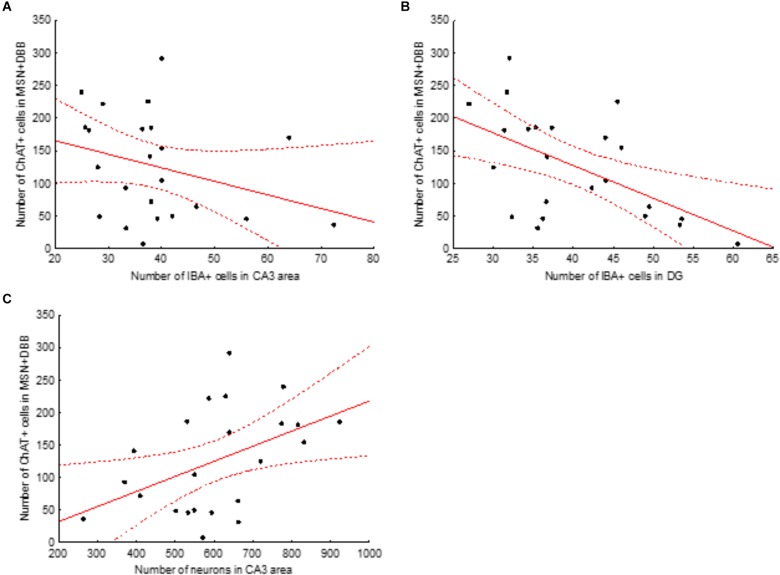
Correlations between the number of ChAT-positive cells in the medial septal nucleus (MSN) and diagonal band of Broca (DBB) area and number of IBA-positive cells in the CA3 area **(A)** and dentate gyrus **(B)** and number of neurons in the CA3 area **(C)**. Solid red line, linear regression curve; dashed lines show 95% confidence intervals. Correlations shown in **(B,C)** are significant (for details, see text).

Next, we examined the association between the intensity of loss of cholinergic neurons and the changes observed in the number of microglial cells and loss of CA3 neurons. To this aim, we calculated the correlation between the total number of ChAT-positive cells in the medial septal nucleus and DBB (MSN+DBB) and the number of microglial cells in the different areas of the dorsal hippocampus. We found no significant correlation between the number of ChAT-positive cells and the number of microglial cells in the CA3 area (*p* = 0.15, β = -0.31, *R*^2^ = 0.09). In contrast, the number of microglial cells in the DG was significantly negatively correlated with the number of ChAT-positive cells in the MSN+DBB (*p* = 0.006, β = -0.55, *R*^2^ = 0.3) (Figure 6). We also found a weak but significant positive correlation between the number of CA3 neurons and the number of ChAT-positive cells in the MSN+DBB (*p* = 0.025, β = 0.465, *R*^2^ = 0.22) (Figure 6).

## Discussion

We found that induction of cholinergic deficit by intracerebroventricular injection of Ig-saporin results in a long-term increase in the number of microglial cells in the dentate gyrus and CA3 area. Presumably, the changes observed in the state of microglial cells were a consequence of activation of microglia that occurred at the early stages after injection of immunotoxin (Rossner et al., 1995b). Our finding that the expression of IL1b, a marker of the acute phase of neuroinflammation, was not altered in all brain structures studied suggests that the altered state of microglia was not a reflection of acute inflammatory process but rather reflected a chronic inflammatory process that occurred after treatment with the immunotoxin. Interestingly, activated microglia and astrocytes (Sastre et al., 2006) and elevated levels of proinflammatory cytokines (Fillit et al., 1991; Strauss et al., 1992) are present in post-mortem AD brains, indicating the involvement of neuroinflammatory processes in the pathogenesis of the disease. Microglia in particular have been implicated in the excessive neuronal loss seen in AD (Varnum and Ikezu, 2012), which is in accordance with the results of the present study. However, we did not find direct correlation between the levels of glial activation and the region-specific degeneration of neurons in the dorsal hippocampus. These data also suggest a possible regional resilience of neurons of the hippocampal fields to the consequences of Ig-saporin action, first of all, and the development of a cholinergic deficit.

A role of the cholinergic system in modulation of memory and hippocampal plasticity via the interaction of acetylcholine with α7 nicotinic acetylcholine receptors located on non-neuronal cells was discussed recently (Maurer and Williams, 2017). The absence of the regulatory effect of acetylcholine may evoke long-term glia activation in the hippocampus. Our data on the correlation between cholinergic cell loss in the MS/DBB area and the number of microglial cells in the DG support the presence of this regulatory mechanism.

We found that microgliosis was associated with neuronal cell death only in the CA3 area but not in other areas of the dorsal hippocampus. This led us to conclude that death of cholinergic neurons in the medial septal area and DBB was the major factor that led to loss of CA3 neurons. The phenomenon of different vulnerability and/or resilience of hippocampal sub-fields to various insults is well known. The most evident effects are the selective vulnerability of CA1 pyramidal neurons to global cerebral ischemia (Herguido et al., 1999) or the selective loss of CA3 pyramidal neurons after seizures induced by kainic acid (Nitecka et al., 1984). In the present study, cell loss was observed in the CA3 pyramidal layer though we did not study its specific mechanism. The other important observation is the increased number of ectopic cells in the CA3 stratum lucidum. This subregion of CA3 is defined as “a-neuronal” (Amaral and Lavenex, 2007). A possible source of neurons in this subregion is migration from the CA3 stratum pyramidale (Lana et al., 2017). Under pathological conditions, such as hypoperfusion or ischemia, activation of cell death-related proteases may result in destruction of the cytoskeleton during the early stages of cell demise, which promotes detachment of neurons from their surrounding cells (Böhm, 2003). Furthermore, these enzymes may be secreted by neurons (Onufriev et al., 2009) and cleave their substrates in the extracellular space, thus facilitating migration of cells to the stratum lucidum.

Our data on the cell loss in the CA3 area may be also considered from the viewpoint of neurodegenerative diseases that are associated with the development of cholinergic deficit. It was shown that, on the one hand, neurons in the CA3 remain practically unaffected by AD (West et al., 1994, 2004) whereas, on the other hand, some AD cases are associated with loss of CA3 neurons (Šimić et al., 1997; Padurariu et al., 2012). It is also known that AD is always associated with a loss of cholinergic neurons in the NBM and only in some cases with a loss of neurons in the medial septum and DBB (Lehéricy et al., 1993). However, so far there are no data on the relationship between the extent of the loss of cholinergic neurons in the medial septum and DBB region and the extent of death of CA3 neurons in AD. Our data suggest that the AD cases associated with degeneration of cholinergic neurons in the areas that project to the hippocampus may also be associated with loss of hippocampal neurons in the CA3 area. DLB is also associated with a loss of cholinergic neurons that project to the hippocampus (Fujishiro et al., 2006), but it seems that this pathology is not associated with loss of CA3 neurons (Harding and Halliday, 2001) and is instead accompanied by accumulation of Lewy bodies in the hippocampal CA2/3 area (Armstrong and Cairns, 2015). The latter as well as our data that the correlation between the level of degeneration of cholinergic neurons in the medial septum and DBB region and degeneration of CA3 neurons suggests that the cholinergic innervation may be important for the viability of CA3 neurons.

It is well known that acute neuroinflammation affects the functioning of vascular cells (Akanuma et al., 2011). We found that 1.5 months after the injection of immunotoxin, which induces acute neuroinflammation, the expression of genes associated with transport functions of endothelial cells decreased in the dorsal hippocampus. Previous data suggest that acute neuroinflammation is associated with an increase in the brain level of prostaglandin E2 (a substrate of Slc22a8) and this increase is associated with a decrease in the level of Slc22a8 (Akanuma et al., 2011). The protein products of two other genes – Ptprb and Sdpr (Cavin-2) – are also involved in the regulation of vascular permeability and transport functions of vascular cells (Hansen et al., 2009; Frye et al., 2015; Hale et al., 2017). Presumably, acute neuroinflammation that occurred at the early stages after Ig-saporin treatment led not only to an acute but also prolonged decrease in the level of Slc22a8. Taken together, the data on the expression of vascular genes suggest that the state of vascular cells 1.5 months after induction of inflammation in the dorsal hippocampus by Ig-saporin was changed and these changes may be associated with altered vascular permeability; however, this effect of Ig-saporin requires further detailed analysis.

Taken together, our data show that the cholinergic deficit induced by Ig-saporin injection is followed by long-term alterations in the hippocampus and some other brain regions. Primarily, this response consisted of a significant increase in the number of microglial cells and their functional activity. This microglial response in the hippocampus was associated with a loss of pyramidal cells in the CA3 field, although our data do not allow us to conclude whether microglia played a pathological or protective role under these conditions. Microglia activation was observed not only in the hippocampus but also in the corpus callosum, indicating generalization of a glial response under the condition of cholinergic degeneration in the medial septal area. Future studies will be important for detailed analysis of the time course and specific functions of microglia after the immunolesion of cholinergic neurons in the basal forebrain.

## Data Availability

All datasets generated for this study are included in the manuscript and/or the Supplementary Files.

## Author Contributions

YD performed intracerebroventricular injections, prepared the brain sections and immunohistochemistry, and wrote the manuscript. MV performed RNA isolation, reverse transcription, and qPCR analysis. AK designed the primers and analyzed qPCR efficiency. TM analyzed immunohistochemical data. NG wrote the manuscript. MS analyzed immunohistochemical data and wrote the manuscript. AM analyzed microglia images. VM designed the study and wrote the manuscript. AB designed the study, fluorescent microscopy, and wrote the manuscript.

## Conflict of Interest Statement

The authors declare that the research was conducted in the absence of any commercial or financial relationships that could be construed as a potential conflict of interest.

## Supplementary Material

FIGURE S1 Immunostaining of choline acetyltransferase in the septal area of control **(A)** and Ig-saporin-treated **(B)** rats. **(C)** Shows the effect of Ig-saporin on the number of ChAT-positive cells in the medial septal nucleus (MSN) + vertical limb of DBB (vDBB) and in the horizontal limb of DBB (hDBB) as well as on total number of cells in both septal areas. All differences between the control and Ig-saporin groups in the respective structures are significant (*p* < 0.003; Mann-Whitney test).

FIGURE S2 Morphology of IBA-stained microglia **(A)** and GFAP-stained astrocytes **(B)** in the hippocampal CA3 area in animals where we observed strong changes in the morphology of microglia, which were accompanied by activation of astrocytes. Scale bar, 40 μm.
