# Supplementary figures and images for: Cholinergic Deficit Induced by Central Administration of 192IgG-Saporin Is Associated With Activation of Microglia and Cell Loss in the Dorsal Hippocampus of Rats
Source: Front Neurosci. 2019 Mar 12;13:146. doi: 10.3389/fnins.2019.00146 (PMC6424051; doi:10.3389/fnins.2019.00146)

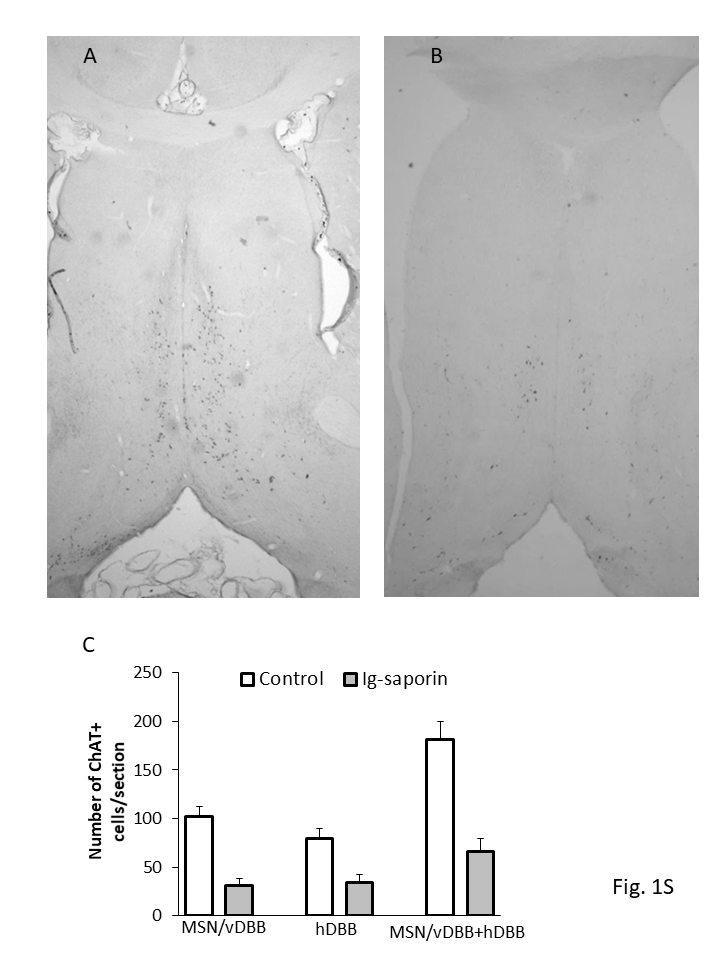

Supplement: FIGURE S1 — Immunostaining of choline acetyltransferase in the septal area of control (A) and Ig-saporin-treated (B) rats. (C) Shows the effect of Ig-saporin on the number of ChAT-positive cells in the medial septal nucleus (MSN) + vertical limb of DBB (vDBB) and in the horizontal limb of DBB (hDBB) as well as on total number of cells in both septal areas. All differences between the control and Ig-saporin groups in the respective structures are significant (p < 0.003; Mann-Whitney test). [file Image_1.TIF]

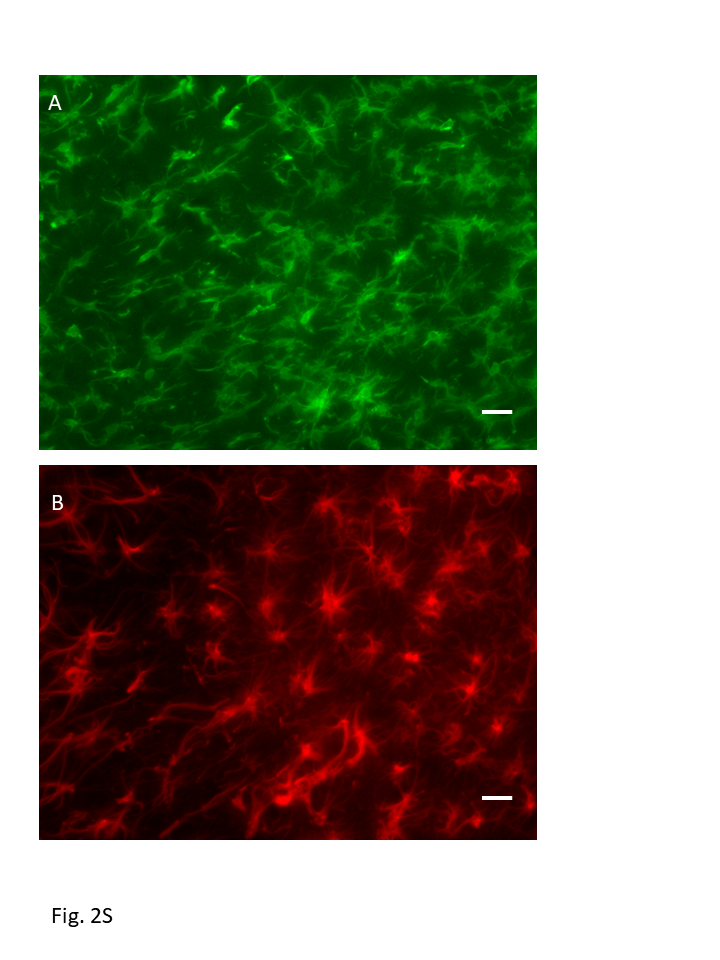

Supplement: FIGURE S2 — Morphology of IBA-stained microglia (A) and GFAP-stained astrocytes (B) in the hippocampal CA3 area in animals where we observed strong changes in the morphology of microglia, which were accompanied by activation of astrocytes. Scale bar, 40 μm. [file Image_2.tif]
